# A Case Report of Rare Coagulation Factor Abnormalities (Factors VII, XI, and XII) in a Patient With Chronic Subdural Hematoma and Treatment With Middle Meningeal Artery Embolization

**DOI:** 10.7759/cureus.79432

**Published:** 2025-02-21

**Authors:** Ryotaro Otsuka, Taro Komuro, Yuto Mitsuno, Satoshi Horiguchi

**Affiliations:** 1 Department of Neurosurgery, Nagahama City Hospital, Shiga, JPN

**Keywords:** acquired coagulation abnormalities, hemophilia, intractable chronic subdural hematoma, middle meningeal artery embolization, neuroendovascular therapy

## Abstract

Acquired coagulopathy is uncommon. Cases of hemophilia A, which is characterized by reduced coagulation factor Ⅷ, have been reported but cases involving diminished factors VII, Ⅺ, or XII have not been reported. We report the case of a patient who presented with a chronic subdural hematoma (CSDH), which was challenging to treat due to underlying acquired coagulation abnormalities. Middle meningeal artery (MMA) embolization is a promising approach for managing refractory CSDH and has proved effective in this case.

An 82-year-old man with no significant medical history and family history presented with a headache and discomfort in his right lower extremity. A left CSDH was detected. Subsequent tests revealed prolonged activated partial thromboplastin time and decreased activities of coagulation factors VII, Ⅺ, and XII. Emergency burr-hole irrigation was performed after administering recombinant coagulation factor VIIa. Two months later, the patient developed aphasia and recurrent hematoma. MMA embolization using N-butyl cyanoacrylate was performed to avoid recurrence, and burr-hole drainage reduced brain compression. The symptoms were relieved, and no recurrence was reported after discharge.

This case description highlights a rare case of CSDH caused by the decreased activities of factors VII, XI, and XII. In addition, the efficacy of MMA embolization in patients with CSDH and limited coagulation disorder treatment options was demonstrated.

## Introduction

Abnormalities in acquired coagulation factors are usually associated with acquired hemophilia, which occurs in 1.48 per one million persons per year in the United Kingdom [1]. Although a few cases of chronic subdural hematoma (CSDH) in patients with factor VII (FVII) deficiency have been reported, reports on other coagulation factor abnormalities are rare. A case involving decreased activity of FVII, factor XI (FXI), and factor XII (FXII) leading to the development of CSDH is unprecedented. CSDH is common and recurs in 10%-20% of cases [2]. Patients taking anticoagulant medication or patients with coagulation abnormalities have a high recurrence rate [3]. Middle meningeal artery (MMA) embolization therapy for refractory CSDH has garnered attention recently. MMA embolization is effective in more than 90% of CSDH cases and reduces the recurrence rate. MMA embolization therapy appears to be particularly effective in patients with CSDH and coagulation abnormalities or patients who cannot discontinue anticoagulant therapy [4-5].

Even after hematoma removal, patients with coagulation abnormalities may have a higher recurrence rate than the general population, often necessitating repeated surgical interventions. Additionally, MMA embolization can help reduce intraoperative hemorrhagic complications. Given these considerations, MMA embolization may be particularly effective in patients with coagulation abnormalities, offering a safer and more sustainable treatment approach.

We are the first to report this rare coagulation factor abnormality pattern with no established treatment methods. MMA embolization therapy was an effective treatment for this patient.

## Case presentation

**Figure 1:**
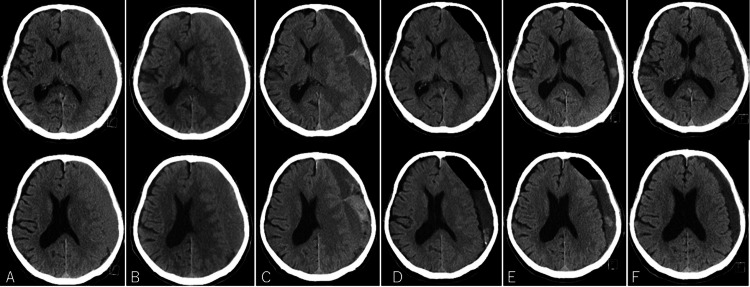
Axial non-enhanced cranial CT images. (A) At the time of the initial visit to our clinic, a CT scan of the head showed a left CSDH, but the degree of compression of the brain by the hematoma was mild. (B) When the patient noticed paralysis in the right lower leg, the cranial CT revealed an increase in the left CSDH. (C) When the patient became unconscious, the left CSDH had further increased, resulting in brain compression and deviation. (D) On the first postoperative day, cranial CT showed that the hematoma had largely been removed, with no brain compression observed. (E) Cranial CT at discharge showed no recurrence of CSDH. (F) Post-discharge cranial CT showed shrinkage of the remaining hematoma. CT, computed tomography; CSDH, chronic subdural hematoma

An 82-year-old man consulted his primary care physician due to a headache and discomfort in his right lower extremity. These symptoms persisted despite a history of overall good health with no significant medical conditions, including no history of hematologic, hepatic, or renal disorders, no prior blood transfusions, and no family history. The patient was not on any medication, including antiplatelet or anticoagulant therapy, and had no history of head trauma or bleeding tendencies, such as gingival or subcutaneous bleeding. After experiencing persistent symptoms for a month, the patient was referred to our neurosurgical outpatient clinic. Physical examination showed equal muscle strength in both lower extremities. A cranial computed tomography (CT) scan revealed a left-sided CSDH (Figure 1A). Despite stable symptoms, imaging revealed a slight increase in the hematoma size two weeks later. Six days later, the patient reported paralysis in the right lower extremity. CT scan revealed further enlargement of the hematoma and a midline shift (Figure 1B). Thus, the patient was admitted to the hospital for a comprehensive evaluation.

Blood tests at admission revealed a prolonged activated partial thromboplastin time (APTT) of 71.7 seconds and a prothrombin time (PT)-international normalized ratio of 1.0. Platelet count, liver function tests (aspartate aminotransferase, alanine aminotransferase, gamma-glutamyl transpeptidase, and alkaline phosphatase), and vitamin K were within the normal range. Tests, including bleeding time, mixing studies, and activated clotting time (ACT), were not performed. The cause of the coagulation disorder was investigated, and emergency surgery was planned if the patient’s condition deteriorated. Coagulation factors VIII and IX and von Willebrand factor levels were normal, and lupus anticoagulant, anticardiolipin IgG antibody, and anti-cardiolipin β2GP I complex antibody tests were negative. However, the activities of FVII, FⅪ, and FXII were decreased. The activity levels of each coagulation factor were as follows: factor II (FII) 79%, factor V 120%, FVII 54%, FVIII 110%, FIX 94%, factor X (FX) 72%, FXI 64%, and FXII 33%. Other coagulation test results were as follows: fibrin degradation product (<2.5 μg/mL) and D-dimer (0.73 μg/mL). No inhibitors to these coagulation factors were detected in additional tests. This pattern of coagulation factor defect has not been reported; thus, no treatment has been established.

On day 6 after admission, the patient’s consciousness was altered (Glasgow Coma Scale [GCS] 12), and the paralysis in his right upper and lower limbs worsened. A CT scan showed significant hematoma expansion, compressing the opposite brain hemisphere. High-intensity areas on the CT images suggested fresh hemorrhage (Figure 1C). Emergency burr-hole irrigation of the hematoma was deemed essential. However, prolonged APTT in blood tests indicated an increased risk of bleeding during surgery. After consulting with a hematologist, recombinant coagulation factor VIIa (NovoSeven®, Novo Nordisk A/S, Bagsværd, Denmark) was administered, and the surgery was performed. Following the administration of NovoSeven, the patient’s APTT improved from 89 seconds to 53 seconds, but clinical bleeding did not show change.

Under local anesthesia, a burr-hole craniotomy was performed at the temporal bone. After incising the hematoma capsule, dark brown blood was ejected. Due to the risk of bleeding from the cerebral surface, irrigation was minimized, and no drainage tube was placed. Although no bleeding from the cerebral surface vessels was observed, managing significant venous bleeding from the subcutaneous tissue was challenging. Hemostasis was achieved by coagulating the bleeding vein and applying pressure through subcutaneous stitches.

Symptoms markedly improved postoperatively. Consciousness was fully restored (GCS 15), and no paralysis in the right upper and lower limbs was detected. Although residual hematoma was detected on CT, the brain was decompressed. No signs of bleeding were detected on follow-up CT (Figure 1D). The patient was discharged home without any sequelae. After hospital discharge, the patient was asymptomatic and lived independently (Figures 1E, 1F). However, two months later, the patient developed aphasia and was taken to the emergency room. CT scans revealed a hyperdense lesion indicative of new bleeding and obscured cerebral sulci that worsened over the previous three weeks (Figure 3A). Suspecting that the symptoms were caused by pressure on the language area or a pressure-induced epileptic seizure, treatments for cerebral edema and epilepsy were initiated. The aphasia symptoms resolved. No curative treatment for the coagulation factor abnormalities exists; therefore, the patient was considered at high risk for future recurrences of CSDH, necessitating a more effective treatment. Consequently, two weeks after admission, embolization of the MMA using N-butyl cyanoacrylate (NBCA) was performed.

Surgical procedure

**Figure 2:**
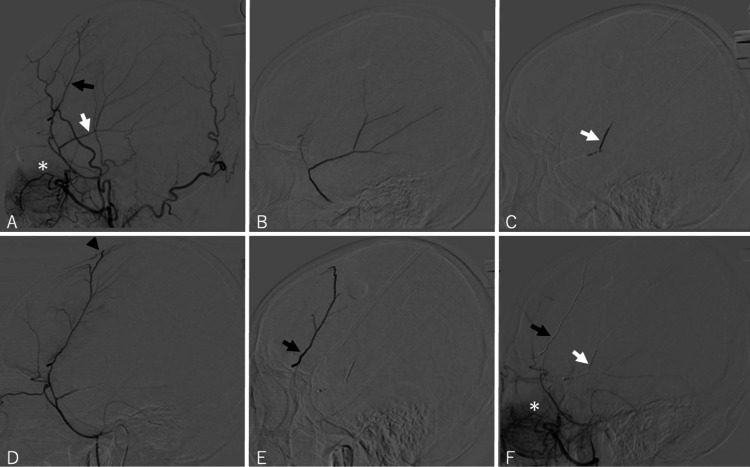
Angiograms in the lateral view. (A) Left ECA angiogram, displaying the ACB (black arrow) and PCB (white arrow), with the RMA marked by an asterisk. (B) Superselective angiogram of the PCB. (C) Injection of NBCA into the left PCB. (D) Superselective angiogram of the ACB, highlighting the hypervascular capsule of the subdural hematoma (black arrowhead). (E) Injection of NBCA into the left ACB. (F) Postoperative angiogram of the left ECA, showing complete occlusion of both the left ACB (black arrow) and PCB (white arrow), with the RMA remaining patent (asterisk). ECA, external carotid artery; ACB, anterior convexity branch; PCB, posterior convexity branch; RMA, recurrent meningeal artery; NBCA, N-butyl cyanoacrylate

Under local anesthesia, a 6-Fr FUBUKI catheter (ASAHI INTECC, Aichi, Japan) was inserted into the trunk of the left external carotid artery (ECA), and a 4.2-Fr FUBUKI catheter (ASAHI INTECC, Aichi, Japan) was inserted into the MMA near the foramen spinosum (Figure 2). Angiography confirmed the location of the recurrent meningeal artery (RMA). We executed the procedure meticulously, considering the NBCA inflow into the RMA, which could potentially anastomose with the ophthalmic artery. A Marathon microcatheter (Medtronic, Minneapolis, MN, USA) was introduced into the left posterior convexity branch (PCB) of the MMA. Selective angiography was performed, and 12.5% NBCA (0.012 mL) was injected, avoiding reflux into the RMA, which may have an anastomosis with the ophthalmic artery. An iED coil (Kaneka, Osaka, Japan) was deployed at the branch’s origin. A separate Marathon microcatheter was used for the left anterior convexity branch (ACB) of the MMA, followed by selective angiography and injection of 12.5% NBCA (0.024 mL). An iED coil was placed at the branch’s origin. The left ACB and PCB were completely occluded, and the RMA was patent, as confirmed by left ECA angiography. No postoperative complications, including visual field defects, were detected.

Due to significant subcutaneous bleeding during the initial surgery and the high bleeding risk from unresolved coagulation abnormalities, we allowed time for postoperative coagulation stabilization and natural hematoma resorption. Since the hematoma did not resolve post-embolization and there was a risk of recurrent aphasia due to brain compression, along with the patient's request, burr-hole drainage was performed on day 9 post-embolization.

**Figure 3:**
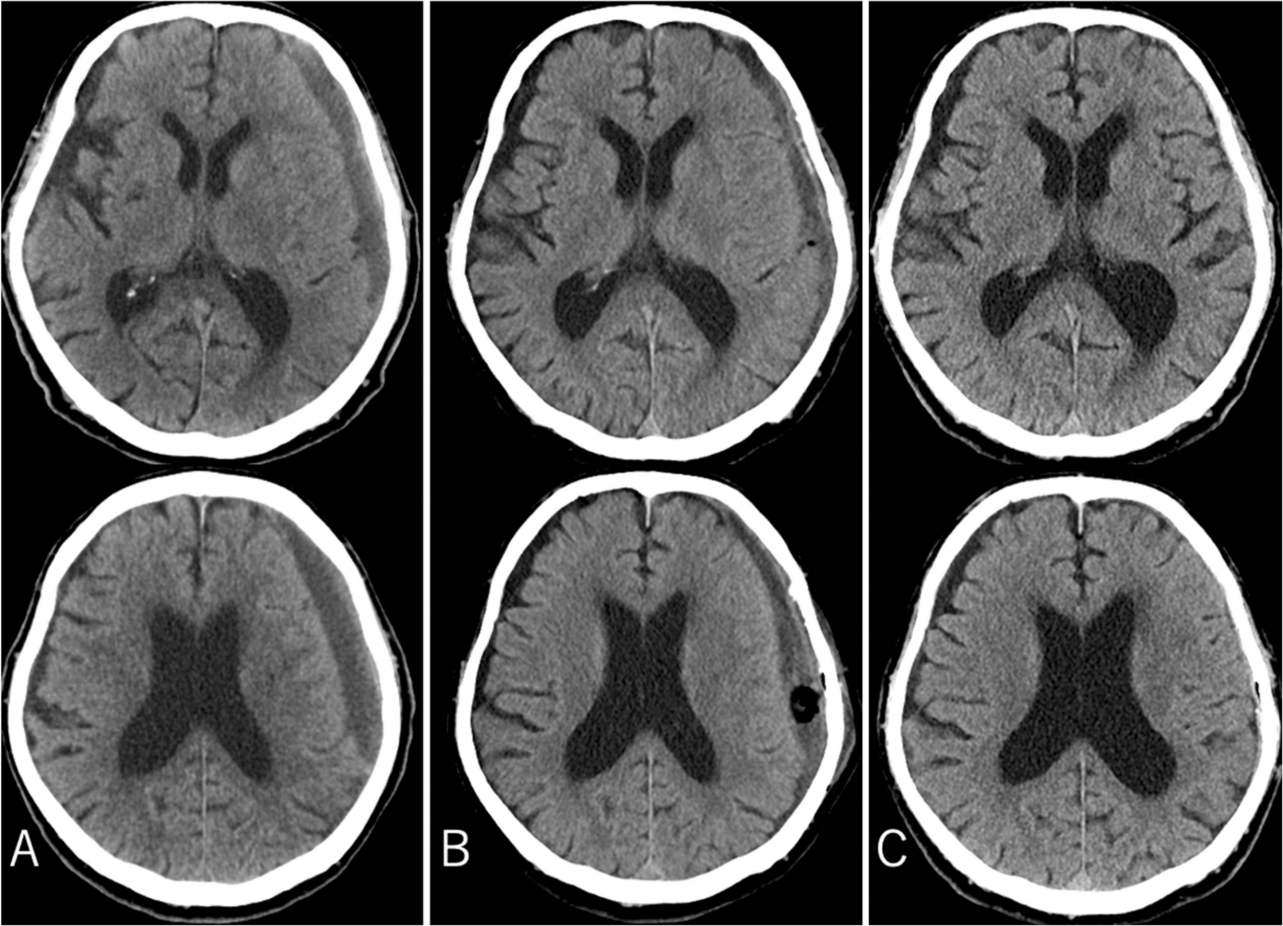
Axial non-enhanced cranial CT images. (A) Two months post-surgery, when the patient developed aphasia, cranial CT revealed a recurrence of the left CSDH. (B) After MMA embolization and a second burr-hole surgery, cranial CT showed that the hematoma had been removed and the cerebral compression had disappeared. (C) The patient has remained asymptomatic for over six months, and a CT scan at six months postoperatively shows no recurrence of CSDH. CT, computed tomography; CSDH, chronic subdural hematoma; MMA, middle meningeal artery

Unlike the first surgery in which hemostasis was difficult, minimal venous bleeding occurred at the skin incision site during the second surgery. The reason for the reduced subcutaneous bleeding compared to the initial surgery remains unclear, but anastomoses between the MMA and subcutaneous blood vessels were considered a possible factor. After surgery, the patient was asymptomatic and discharged. No hematoma recurrence was detected by CT at the time of discharge (Figure 3B). The patient has been recurrence-free for more than six months since discharge (Figure 3C).

## Discussion

In our case, the patient presented with bleeding symptoms for the first time at the age of 82. Given the absence of a bleeding tendency from birth until this age, and the exclusion of hemophilia A, hemophilia B, and von Willebrand disease, which constitute the majority of congenital coagulation disorders, a congenital condition is unlikely. We diagnosed a rare, acquired coagulation disorder. The following sections describe the pathophysiology and treatment of acquired coagulation factor abnormalities.

Pathogenesis of acquired coagulation abnormalities and CSDH

The most common acquired blood coagulation disorder is acquired hemophilia, characterized by sudden bleeding due to the inhibition of coagulation factors VIII and IX. Acquired hemophilia A is caused by inhibitors of FVIII and occurs in 1.4 per one million individuals per year in the UK [1]. Bleeding due to inhibitors of other coagulation factors is rare. In this case, the patient with CSDH showed marked APTT prolongation and moderately decreased activity of coagulation factors VII, Ⅺ, and Ⅻ.

No inhibitors were detected against any coagulation factors. The detection rates of acquired inhibitors against FVIII and FIX, which are the most commonly affected factors in acquired coagulation disorders, are 30% and 1-3%, respectively, indicating a relatively low occurrence [6]. Detection rates of acquired inhibitors against other coagulation factors have not been reported, and thus their prevalence remains unclear.

Acquired FXII coagulation abnormality

According to previous reports, acquired abnormal coagulation of FXII does not cause bleeding, even in cases of complete deficiency [7]. FXII deficiency is generally not associated with clinical bleeding, as it primarily affects the contact activation pathway without significantly impairing hemostasis. Given the normal PT and the absence of reported bleeding tendencies in FXII-deficient individuals, we consider FXII deficiency in this case to be incidental rather than contributory.

Acquired FVII coagulation abnormality

Acquired coagulation abnormality of FVII has been associated with the use of anti-thymocyte globulin in aplastic anemia, as well as with penicillin, cephalosporins, interleukin-2, malignancies, and conditions such as sepsis, pancreatitis, and multiple myeloma [7]. Abdulsalam et al. reported a severe case of intracranial hemorrhage with prolonged PT in a patient with systemic lupus erythematosus [8]. The recommended treatment modalities include recombinant factor VIIa and factor VII inhibitor bypassing activity. However, fresh frozen plasma is less effective [7].

In this case, APTT was markedly prolonged, and the activity of coagulation factors VII, Ⅺ, and XII was moderately decreased. Although decreased activity of coagulation factor VII can lead to a hemorrhagic lesion, the PT should be prolonged; however, the APTT is normal in most cases. The presence of marked APTT prolongation suggests the involvement of factors beyond FVII.

Acquired FⅪ coagulation abnormality

After being activated by FXIIa, FXIa, also known as plasma thromboplastin antecedent, contributes to the intrinsic pathway of blood coagulation by activating factor Ⅺ. Acquired inhibitors to FXI are associated with systemic lupus erythematosus and malignancies. Patients present with or without bleeding manifestations, but the APTT is typically prolonged [7]. FXI is a promising anticoagulation therapy target due to its minimal impact on normal hemostasis and its potential to reduce bleeding complications better than other anticoagulants [9]. Interestingly, although FXI minimally impacts normal hemostasis, FXI-deficient patients may experience bleeding after trauma or surgery in regions of high enzymatic thrombolysis (e.g., mucosa of the mouth and nose, urinary tract, gastrointestinal mucosa, obstetric tissues, surgical sites, and post-traumatic sites) [10].

The research analyzing the hematoma contents from CSDH has identified an increase in fibrinolytic factors, implicating their role in the onset and progression of CSDH [11]. Additionally, cerebral vessels are affected by FXI deficiency more than cardiovascular vessels. Precursor amyloid β protein may accumulate in cerebral vessels with age; when proteolyzed, the precursor amyloid β protein produces amyloid β protein with fibrinolytic activity. This specific phenomenon, which is not detected in the cardiovascular system, implies that cerebral vessels, especially in older adults, are more susceptible to FXI deficiency than cardiovascular vessels [12].

From the foregoing, it can be inferred that patients with diminished FXI activity are predisposed to developing CSDH, particularly older individuals with cerebral atrophy following trauma. These observations are consistent with the current case. The patient did not exhibit spontaneous bleeding lesions in areas other than the cerebral vessels, such as subcutaneous hemorrhages. To our knowledge, the coagulation abnormalities in our patient have not been previously reported nor has the onset of CSDH under these conditions been reported.

There is no established treatment for decreased FⅪ activity. The use of fresh frozen plasma and recombinant factor VIIa and factor VII inhibitor bypassing activity has been suggested, but their effectiveness is unclear [7]. Vazzana et al. reported the case of an elderly patient with acquired FⅪ inhibitor presenting with bilateral subdural hematomas [13]. The patient had a history of cancer and multiple blood transfusions, which may have contributed to the development of the inhibitor. The patient did not undergo surgical treatment but was treated with anti-cerebral edema drugs and ultimately died.

Treatment of refractory and intractable chronic subdural hematoma

CSDH recurs in 10%-20% of cases. Factors reported to increase the risk of recurrence include postoperative midline shift (≥5 mm), diabetes mellitus, preoperative seizure, preoperative hematoma width (≥20 mm), anticoagulant therapy, terminal malignancy, old age, and postoperative residual air (>20%) [2,14]. The fact that antiplatelet agents are not a risk factor for recurrence does not contradict the pathophysiology of CSDH [3,11]. Refractory CSDH has recently been treated with MMA embolization. MMA embolization, utilizing embolic materials such as NBCA, Onyx, or coils, is intended to reduce blood supply to the neovasculature of the CSDH membrane by occluding the MMA’s anterior and posterior branches (ACB and PCB), thereby preventing recurrence. The recurrence rate after MMA embolization is less than 5%, with a complication rate of around 4% [4]. The procedure is effective in 91% of cases, and hematoma volume is reduced after MMA embolization alone in 70% of cases [4-5]. The rate of complete hematoma resolution is higher after the embolization of both branches compared with the embolization of a single branch (76% vs. 33%, p = 0.014) [15]. Based on the lack of definitive curative treatments for the coagulation abnormalities in our patient, the high likelihood of recurrent CSDH, and the risks associated with frequent surgical interventions leading to hemorrhagic complications, MMA embolization was considered the most effective method for treating the CSDH. NBCA was used to completely occlude both the anterior and posterior branches of the MMA to increase the efficacy. The procedure was safe, without any complications. The effectiveness of the treatment is demonstrated by the absence of recurrence. While MMA embolization in pediatric patients with hemophilia A for acute subdural hematoma has been reported [16], this is the first report of MMA embolization in a patient with CSDH and abnormalities in FVII, FXI, and FXII.

## Conclusions

This case report is important for several reasons. First, an uncommon occurrence of CSDH triggered by reduced activities of factors VII, XI, and XII I is documented. Second, this report delves into the pathophysiology of coagulation factors as they relate to CSDH. Lastly, this report highlights the efficacy of MMA embolization in patients lacking effective coagulation disorder treatments. Thus, this report advances medical knowledge.
